# Survival Without Reintervention of Second Artificial Urinary Sphincter Implants in Men: A National Healthcare Data System-Based Study in France

**DOI:** 10.1590/S1677-5538.IBJU.2025.0374

**Published:** 2025-08-30

**Authors:** Elliot Tokarski, Yoann Taillé, Emmanuel Chartier-Kastler, Bertrand Lukacs, Thomas Seisen, Morgan Roupret, Aurélien Beaugerie, Eric Vicaut, Pierre C. Mozer, Louis Lenfant

**Affiliations:** 1 Sorbonne University Pitié-Salpêtrière University Hospital Department of Urology Paris France Department of Urology, Pitié-Salpêtrière University Hospital, Sorbonne University, AP-HP, Paris, France; 2 Paris Cité University Saint Louis-Lariboisière-Fernand Widal Hospital, AP-HP Clinical Research Department Paris France Clinical Research Department, Saint Louis-Lariboisière-Fernand Widal Hospital, AP-HP, Paris Cité University, Paris, France; 3 Health Data Hub, AP-HP Paris France Health Data Hub, AP-HP, Paris, France

**Keywords:** Urinary Incontinence, Urinary Sphincter, Artificial, Retrospective Studies

## Abstract

**Purpose:**

Artificial urinary sphincter (AUS) is the gold standard treatment for severe male stress urinary incontinence (SUI). While survival outcomes after primary implantation are now well established, the prognosis following reintervention remains poorly understood. We aimed to assess long-term reintervention-free survival after a second AUS implantation and to compare outcomes between device replacement and reimplantation after removal.

**Materials and Methods:**

We performed a nationwide, population-based, retrospective cohort study including all men aged ≥18 years in France who underwent a second AUS implantation between 2006 and 2018 for SUI following prostate cancer or benign prostatic hyperplasia treatment. AUS procedures were identified through a unique device identifier. Of 5,132,311 eligible men, 8,475 received a first AUS and 1,619 a second AUS: 1,165 after device replacement and 454 after reimplantation following removal. The primary outcome was reintervention-free survival, estimated by Kaplan–Meier analysis. Secondary outcomes included replacement and removal rates. Predictors of reintervention were identified using multivariable Cox regression.

**Results:**

Median follow-up was 53 months (IQR 26–81). Reintervention-free survival after second AUS was 81% (95% CI 79–83) at 2 years, 68% (95% CI 65–71) at 5 years, and 61% (95% CI 57–64) at 10 years. Device replacement achieved significantly better survival than reimplantation after removal (p < 0.001). Notably, only 21% of patients whose first AUS was removed underwent reimplantation.

**Conclusions:**

Second AUS implantation provides durable long-term outcomes, approaching those of primary implants. The indication for reintervention critically influences prognosis, with replacement outperforming reimplantation after removal. The low reimplantation rate after AUS removal provides a clinically relevant piece of information to counsel patients requiring device removal.

## INTRODUCTION

Artificial urinary sphincter (AUS) implantation is the gold standard treatment for severe male stress urinary incontinence (SUI) (1), typically secondary to radical prostatectomy (2) for prostate cancer or surgery for benign prostatic hyperplasia. Although the AUS is widely recognized as a reliable and durable device (3, 4), several studies have shown that a significant proportion of patients require reintervention, defined as device removal or replacement, after the initial implantation (5). Reported reintervention rates reach 29% at 2 years and 40% at 10 years according to a French national health care database study (6), 22% at 5 years and 33% at 10 years according to the PIF database in the US (7), and 34% at 10 years in a Canadian study using the Ontario Health Insurance Plan database (8).

To date, the literature has largely focused on reinterventions following primary AUS implantation (3-6, 9), including evidence from a large, prospective, multicenter European cohort (10, 11). In contrast, data regarding the durability of a second AUS, specifically its survival without subsequent surgical revision, remain scarce.

Evidence regarding outcomes after secondary AUS implantation remains conflicting. While one study of 324 patients undergoing replacement found similar survival durations between first and second devices in non-irradiated patients, suggesting that patients might benefit from replacement (12), other reports indicate increased risk. Hebert et al. observed a higher likelihood of device removal due to infection or erosion after replacement in 281 patients and an even greater risk following reimplantation after a first removal in 69 patients (5, 13). Similarly, Lai et al. reported a fourfold increase in urethral erosion after second implantations following prior removals, whereas simple replacements were not associated with elevated erosion or reoperation rates (14). Taken together, these studies provide conflicting evidence regarding outcomes after AUS replacement. While some suggest acceptable durability, others indicate heightened risk, highlighting the need for robust, population-based studies to better assess outcomes and prognostic factors following secondary AUS implantation in real-world settings.

We therefore hypothesized that survival without reintervention for second AUS would differ according to the indication for reintervention. Specifically, we expected replacement to be associated with better survival than reimplantation after removal, given the potential for urethral compromise following removal, and that factors such as prior radiotherapy or non-prostatectomy pelvic surgery would be associated with reduced device survival.

Our study aimed to assess survival rates after a second AUS implantation. Specifically, we aimed to investigate differences in outcomes between patients who underwent removal followed by reimplantation and those who received a simple replacement. Indeed, device removal is often associated with urethral injury or erosion, and subsequent implantation could be at a higher risk of surgical reintervention. Additionally, we seek to identify prognostic factors associated with reduced survival of the second device, examining whether these factors, such as prior radiotherapy or surgeries other than prostatectomy, impact outcomes. These findings are intended to enhance clinical decision-making and improve the management of patients requiring AUS reintervention.

## MATERIALS AND METHODS

### Study design

This retrospective, population-based cohort study was conducted using the Observapur database ("*OBSERVAtoire de la Prise en charge en URologie*"), derived from the French National Health Data System (SNDS). The SNDS includes hospital discharge data (Programme de Médicalisation des Systèmes d'Information, PMSI) and outpatient healthcare reimbursement claims (*Système National d'Information Interrégimes de l’Assurance Maladie, SNIIRAM*), enabling comprehensive, longitudinal tracking of diagnoses, procedures, and medical devices in all healthcare sectors in France. The methodology underpinning the database and its application in urologic research has been previously described in detail by Lenfant et al. (6). The study was approved by the French Data Protection Authority (C*ommission Nationale de l'Informatique et des Libertés, CNIL DE-2010-002*), and conducted in compliance with European data protection regulations.

All male patients aged ≥18 years who underwent a first AUS implantation between 2006 and 2018 were identified using specific device reimbursement codes (LPP 3121402) and surgical procedure codes (CCAM JELA002) (15). All AUS-related events, including initial implantation, replacements, removals, and second device implantation, were recorded through December 31, 2018.

In France, the AMS 800™ is the only AUS reimbursed by the national health insurance system under the LPP code 3121402. Therefore, all procedures included in this study exclusively involved the AMS 800™ device.

The present study focused on patients who received a second AUS, defined either as a replacement of a previously implanted AUS or a new implantation following complete removal of the initial device.

### Definition of second AUS survival

Two distinct clinical trajectories defining the "second AUS survival" were identified. 1) Post-replacement survival: For patients who underwent a replacement of the initial AUS, survival was defined from the date of the first replacement until either a subsequent replacement or complete device removal. 2) Post-Reimplantation Survival: For patients who underwent removal followed by a new AUS implantation, survival was measured from the reimplantation date until the second device's replacement or removal (see Figure-S1).

### Outcomes

The primary outcome was reintervention-free survival following second AUS implantation, defined as the interval from the second implantation (via replacement or reimplantation) to either device removal or subsequent replacement.

Secondary outcomes included the separate assessment of survival until removal of the second AUS and survival until replacement of the second AUS. Secondary outcomes also included survival without reintervention stratified according to the indication for the second AUS (replacement vs. removal followed by reimplantation).

Baseline covariates included age, preexisting medical conditions (including BPH, PCa), comorbidities (diabetes, hypertension, obesity, chronic obstructive pulmonary disease), and concurrent medication use (antiplatelets, anticoagulants, antimuscarinics). Diagnoses were identified using ICD-10 codes while surgical and procedural interventions were recorded using CCAM and LPP codes. The potential risk factors evaluated for reintervention were identical to those analyzed in the initial AUS survival study (6).

### Statistical analysis

Descriptive statistics were reported as medians with interquartile ranges (IQR) for continuous variables, and frequencies with percentages for categorical variables. Kaplan-Meier estimates were used to calculate survival probabilities from the date of second AUS implantation to reintervention, removal, or replacement. Comparative survival analyses were stratified according to the etiology of male SUI, the annual implantation volume, and the indication for the second AUS (replacement vs. removal followed by reimplantation). Associations between patient- and center-level characteristics and reintervention risk were assessed using multivariable Cox proportional hazards models, with clinically relevant covariates selected a priori. Proportional hazards assumptions were tested using Schoenfeld residuals.

All statistical tests were two-sided, with a significance threshold of p < 0.05. Analyses were performed using R version 4.2.2 (R Foundation for Statistical Computing, Vienna, Austria).

## RESULTS

**Figure 1:**
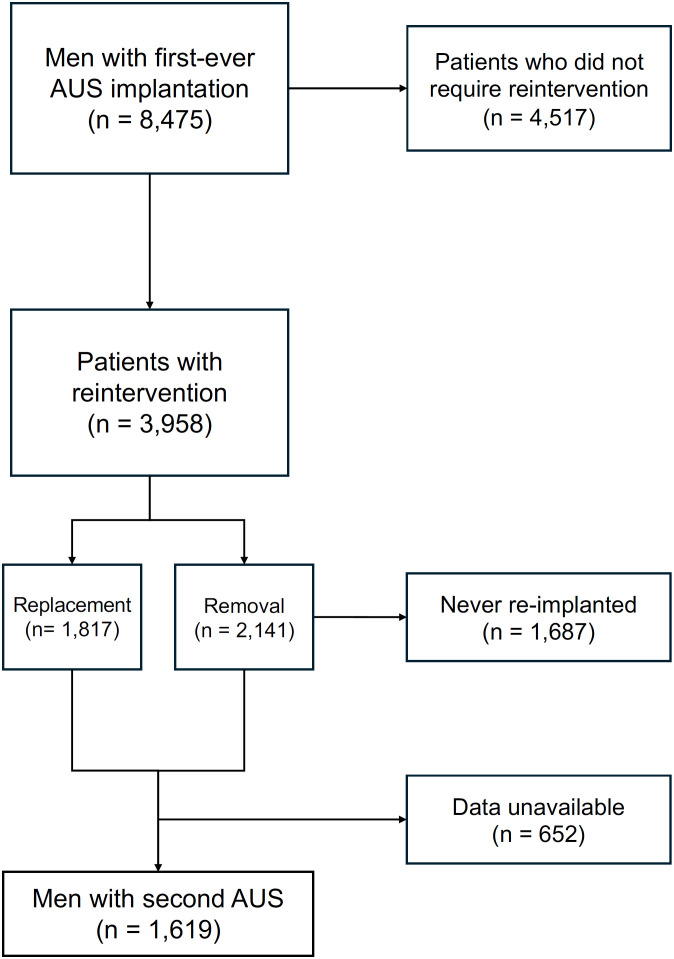
Study cohort selection process of men aged 18 years and older with benign prostatic hyperplasia or prostate cancer who had a second artificial urinary sphincter implantation in France between 2006 and 2018.

Between 2006 and 2018, 8,475 patients were included in the first survival analysis. Of these, 3,958 underwent reintervention, 2,141 underwent removal, and 1,817 underwent replacement. After removal, 454 of 2,141 patients (21%) received a second AUS. Among patients who had a replacement of a first AUS, second survival data were available for 1,165 individuals. In total, 1,619 men who underwent a second implantation of an AUS (AMS 800) between 2006 and 2018 were included in the analysis (Figure-1). The median follow-up after second AUS implantation was 53 months (IQR, 26-81).

The median age at the time of the second implantation was 71 years (IQR, 66-76). Common comorbidities included diabetes in 23% of patients, obesity in 22%, chronic obstructive pulmonary disease (COPD) in 22%, and active smoking in 12% of patients at the time of their second implantation (Table-1).

**Table 1:** Population characteristics.

Characteristic		Overall	
		N = 1,6191	
Age - years (IQR)		71.0	(66.0, 76.0)
Follow-up - months (median)		53.0	(26.0, 81.0)
**Cause for first reintervention— no. (%)**			
	Explantation	454	(28%)
	Replacement	1,165	(78%)
**BPH surgical treatment — no. (%)**			
	No BPH surgery	1,392	(86%)
	Bladder neck incision	8	(0.5%)
	Laser endoscopic surgery	1	(<0.1%)
	Simple prostatectomy	28	(1.7%)
	Transurethral needle ablation	3	(0.2%)
	TURP	187	(12%)
**Prostate cancer surgical treatment — no. (%)**			
	No Prostate cancer surgery	605	(37%)
	Ablatherm	20	(1.2%)
	Brachytherapy	3	(0.2%)
	Laparoscopic radical prostatectomy	482	(30%)
	Open radical prostatectomy	508	(31%)
	Perineal radical prostatectomy	1	(<0.1%)
Radiation therapy — no. (%)		166	(10%)
**Prior urethra or bladder neck surgery — no. (%)**			
	No prior urethra or bladder neck surgery	1,272	(79%)
	Bladder Neck Incision	159	(9.8%)
	Urethral Stenosis Surgery	175	(11%)
	Urethro-rectal Fistula Surgical Treatments	8	(0.5%)
	Urethroplasty	5	(0.3%)
Diabetes - no. (%)		375	(23%)
Obesity- no. (%)		352	(22%)
Preoperative Tobbaco use - no. (%)		187	(12%)
Preoperative COPD - no. (%)		352	(22%)
Perioperative oral anticoagulant therapy - no. (%)		102	(6.3%)
Perioperative heparine therapy - no. (%)		378	(23%)
Perioperative Antiplatelet therapy - no. (%)		373	(23%)
Perioperative Antimuscarinic therapy - no. (%)		334	(21%)
De Novo Antimuscarinic therapy - no. (%)		334	(21%)
Preop Antimuscarinic therapy - no. (%)		743	(46%)
Perioperative Hormone deprivation therapy - no. (%)		198	(12%)
**Center experience - no. (%)**			
	1 year	110	(6.8%)
	2-5 year	340	(21%)
	6-10/year	281	(17%)
	10-20/year	434	(27%)
	>20/year	454	(28%)

**Figure 2:**
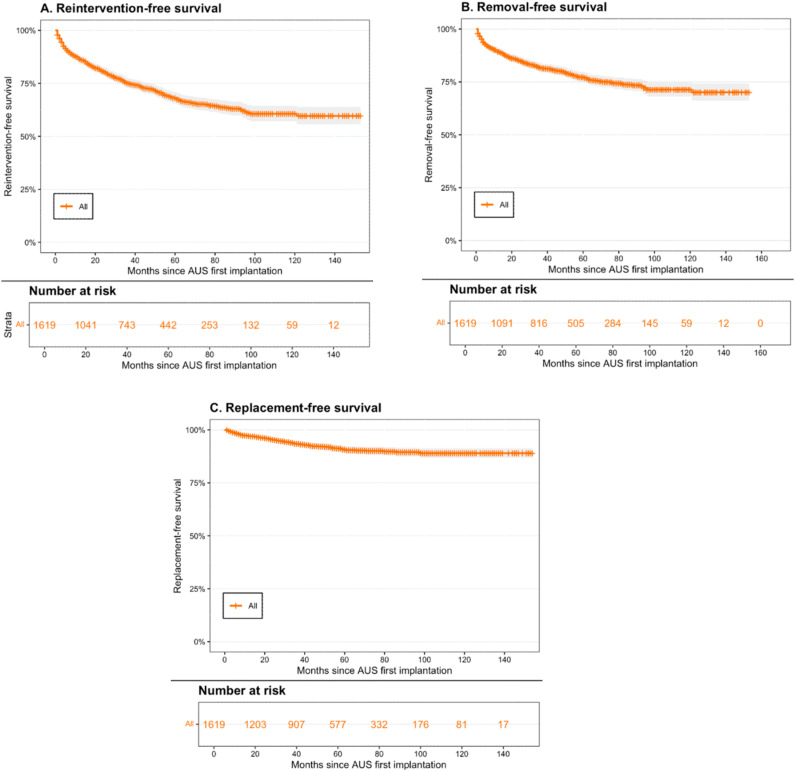
Artificial urinary sphincter reintervention (A), removal (B), and replacement-free survival (C), after second artificial urinary sphincter implantation in 1619 men.

Overall, reintervention-free survival rates of a second AUS were estimated at 81% (95% CI [79%-83%]) at 2 years, 68% (95% CI [65%-71%]) at 5 years, and 61% (95% CI [57%-64%]) at 10 years (Figure-2). During follow-up, 435 patients (27%) required at least one subsequent reintervention. Device removal-free survival rates of a second AUS were 85% (95% CI [83%-87%]) at 2 years, 77% (95% CI [75%-80%]) at 5 years, and 71% (95% CI [68%-75%]) at 10 years, corresponding to a removal rate of 20% (319/1,619) during follow-up. Replacement-free survival rates of a second AUS were 95% (95% CI [94%-96%]) at 2 years, 91% (95% CI [89%-92%]) at 5 years, and 89% (95% CI [87%-91%]) at 10 years, with an overall replacement rate of 7% (116/1,619).

AUS = indicates artificial urinary sphincter.

Survival outcomes differed significantly according to the indication for the second AUS (replacement vs. removal followed by reimplantation). Patients who underwent reimplantation after removal of the first device had significantly lower reintervention-free survival than those who underwent replacement only (p < 0.001) (Figure-3A). At 5 years, only 50% of patients in the removal/reimplantation group remained free from reintervention.

**Figure 3A:**
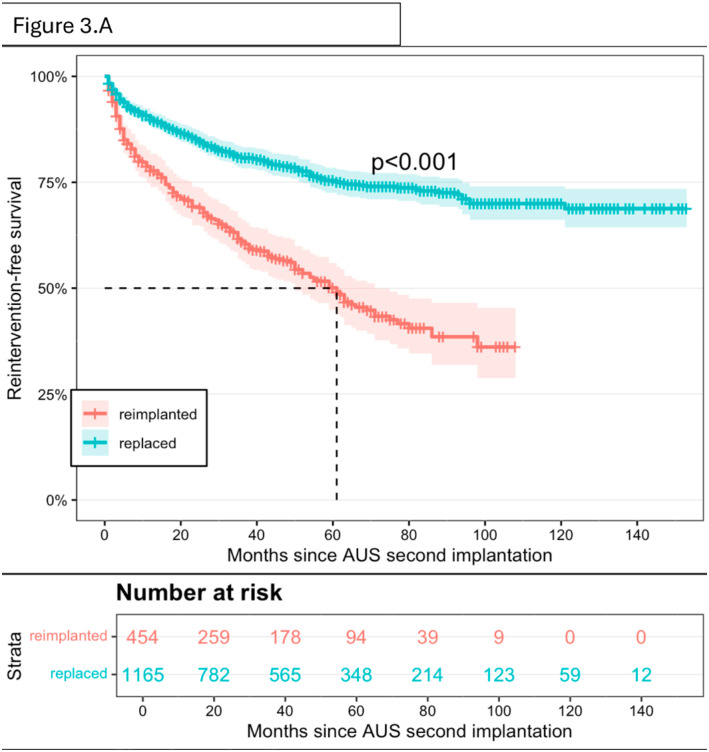
Artificial urinary sphincter reintervention-free survival after second AUS in men according to the cause of first survival end.

AUS = indicates artificial urinary sphincter.

Additionally, the etiology of incontinence had a significant impact on reintervention-free survival. At 2 years, reintervention-free survival was 74% (95% CI [69%-80%]) in patients with a history of BPH surgery, compared to 83% (95% CI [80%-85%]) in those with post-prostatectomy incontinence (p < 0.001), including patients with prostatectomy alone or after prostatectomy with adjuvant radiotherapy (Figure-3B). Pairwise survival analyses were performed across the three groups. Reintervention-free survival was significantly lower in patients with prior BPH surgery compared to those with incontinence after prostatectomy alone or after prostatectomy with adjuvant radiotherapy (RP + RT) (p<0.001 for both comparisons). However, no significant difference was found between the RP + RT group and the prostatectomy-alone group (p =0.09)

**Figure 3B:**
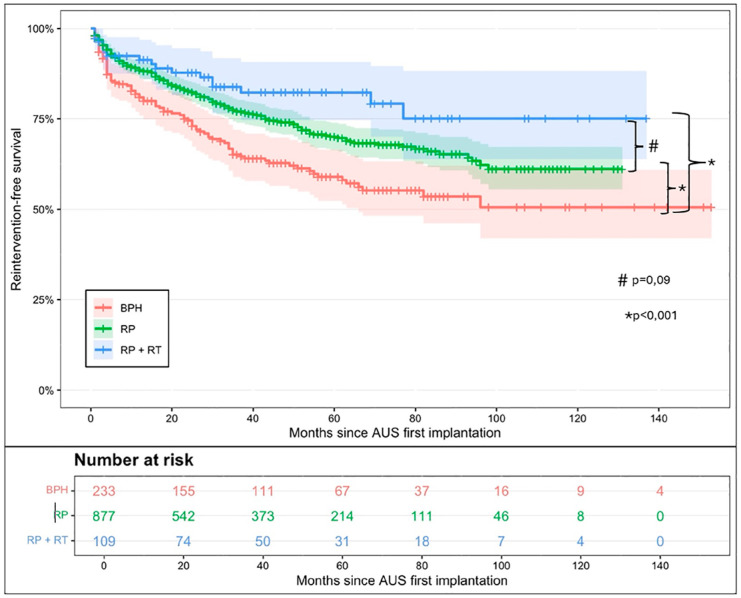
Artificial urinary sphincter reintervention-free survival after second AUS in men according to the cause of male stress urinary incontinence.

AUS indicates artificial urinary sphincter; BPH, benign prostatic hyperplasia; RP, radical prostatectomy. Pairwise survival analyses were performed between the three groups. Reintervention-free survival was significantly lower in patients with prior BPH surgery compared to those with incontinence after prostatectomy alone or after prostatectomy with adjuvant radiotherapy (RP + RT) (p<0.001 for both comparison). However, no significant difference was found between the RP + RT group and the prostatectomy-alone group (p =0.09).

Survival analyses stratified by center annual volume demonstrated a trend toward reduced reintervention-free survival in low-volume centers, although the association did not reach statistical significance when the annual center volume was categorized as 1/year, 2–5/year, 6–10/year, 11–20/year, and >21/year (see Figure-S2). In contrast, replacement-free survival differed significantly across annual volume categories (p = 0.03, see Figure-S3), with an increased replacement rate observed in centers performing fewer AUS implantations annually.

**Figure 4:**
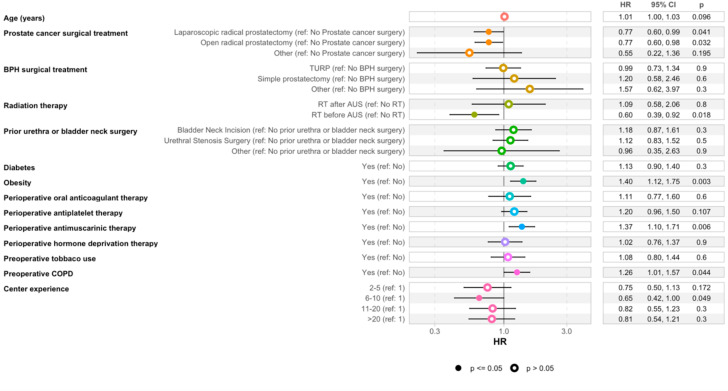
Risk of reintervention after second artificial urinary sphincter implantation according to patient and hospital factors. AUS indicates artificial urinary sphincter; BPH, benign prostatic hyperplasia; COPD, chronic obstructive pulmonary disease.

Multivariate analyses using Cox proportional hazards model indicated that several factors were independently associated with decreased reintervention-free survival following second AUS implantation including obesity (HR = 1.40, [95% CI 1.12-1.75], p = 0.03), antimuscarinic treatment (HR = 1.37, [95% CI 1.10-1.71], p = 0.06) and preoperative COPD (HR = 1.26, [95% CI 1.01-1.57], p = 0.04) (Figure-4). Although not statistically significant, higher annual surgical volume was associated with a trend toward reduced hazards of reintervention after second AUS. Specifically centers performing 6–10 procedures per year demonstrated a statistically significant reduction in reintervention risk compared to centers performing only 1 procedure per year (HR = 0.65, 95% CI [0.42–1.00], p = 0.05).

RP = radical prostatectomy.

## DISCUSSION

AUS implantation remains the gold standard treatment for severe male stress urinary incontinence, yet long-term outcomes are frequently compromised by the need for surgical reintervention. While many studies have addressed the outcomes following primary implantation, limited evidence exists regarding the durability of a second AUS, whether performed as a replacement or as a reimplantation after prior device removal. Our study, leveraging a nationwide French administrative dataset, provides novel insights into the real-world survival of second AUS procedures.

Our study demonstrates that second AUS procedures maintain acceptable long-term durability, with reintervention-free survival rates of 81% at 2 years, 68% at 5 years, and 61% at 10 years. These results are consistent with previously published data. A recent study reported a 29% reintervention rate within two years following initial AUS placement (6). Similarly, Raj et al. (2005) observed comparable 5-year device survival between primary and secondary AUS implantations, supporting the feasibility of replacement strategies. Interestingly, our cohort exhibited an even lower 2-year reintervention rate of 19% following second AUS implantation (16). Comparative data from Hebert et al. (2021), who analyzed 1,360 AUS procedures at the Mayo Clinic, reported significantly worse 1- and 5-year survival following replacement procedures (n=281) compared to primary implants (n=1,079) (90% vs. 85% at 1 year, and 74% vs. 61% at 5 years, p < 0.001) (5). While their findings suggest a decreased durability of the second AUS, they partially contrast with the present results, where second AUS procedures demonstrated survival outcomes comparable to those reported for primary implantations.

Importantly, the outcomes of second AUS device were significantly influenced by the indication for surgery. Patients undergoing replacement achieved a 2-year reintervention-free survival of 85%, whereas those undergoing reimplantation after complete device removal had markedly lower survival rates, with only 50% remaining free from reintervention at 5 years (Figure-3A). This distinction is consistent with findings from the literature. Lai and Boone (2012) reported a fourfold increased risk of urethral erosion following reimplantation compared to primary AUS placement (14). Furthermore, Raj et al. (2006) specifically examined outcomes after AUS reimplantation following prior cuff erosion and observed a second erosion rate of 34.8%, with a mean erosion-free interval of only 6.7 months (17). These results underscore the challenges associated with reimplanting an AUS in a previously compromised urethra. Additionally, comorbidities such as hypertension, coronary artery disease, and prior radiation therapy were significantly more prevalent in this high-risk population, aligning with our findings on the impact of patient-related factors (e.g., obesity, COPD) on second AUS survival. We hypothesize that, compared to patients undergoing device replacement, those undergoing reimplantation after previous device removal may present with more comorbidities and, more importantly, a more fragile urethra, often compromised by prior AUS removal in the context of erosion (18). Future research should aim to directly compare these two groups to better understand the observed survival disparity.

A novel and clinically important finding of our study is the remarkably low rate of reimplantation after AUS removal. Only 21% (454/2,141) of patients who had an AUS removal subsequently underwent reimplantation. To our knowledge, this reimplantation rate has not been previously reported in the literature. Its accurate estimation was made possible by the longitudinal follow-up and the absence of any lost-to-follow-up patients in our study. Further research is ongoing to investigate the factors predicting whether a patient will eventually undergo reimplantation. This information is critical for patient counseling, as it emphasizes that removal often results in definitive loss of AUS candidacy, with reimplantation offering only limited prospects for success.

Finally, we observed that the etiology of incontinence influenced second AUS survival, consistent with previous findings (6). Patients with incontinence secondary to BPH surgery had worse outcomes compared to those with post-prostatectomy incontinence, with a 2-year reintervention-free survival of 74% versus 83%, respectively (Figure-3B). No significant differences were observed between patients with post-prostatectomy incontinence alone and those who had received adjuvant radiotherapy (log-rank p = 0.09). In addition, while not all comparisons reached statistical significance, there was a trend toward improved outcomes in high-volume centers, with a significantly lower reintervention risk observed in centers performing 6–10 AUS procedures per year (HR = 0.65, p = 0.05), suggesting a potential volume-outcome relationship that warrants further investigation (Figure-4).

To our knowledge, this is the largest population-based analysis specifically addressing second AUS survival. The use of the SNDS enables exhaustive patient inclusion and real-life follow-up across all healthcare settings, enhancing generalizability of our findings (19). Moreover, the detailed stratification by indication (replacement vs. reimplantation), etiology, and center volume provides clinically relevant insight that are rarely captured in single-center series.

Several limitations must be acknowledged. First, the SNDS lacks granular clinical data on replacement indications, such as mechanical failure or recurrent incontinence, which precludes cause-specific survival analyses. However, given the scale of our cohort and the extended duration of follow-up, our primary outcome (reintervention-free survival) remains clinically meaningful and highly relevant.

In addition, the method used to create the database based on CCAM procedures means that we only have access to surgical outcomes, which prevents us from obtaining functional results such as continence or patient satisfaction data. We can look forward to future work coupling a database of this design with an analysis of patient records, using national cohort tools to collect data on satisfaction, quality of life and continence, as has been done in neurology (20). Although reintervention-free survival is already clinically meaningful, especially to counsel patients about their risk of needing a third surgery, depending on the time and reason for their reintervention.

Future research should investigate survival and outcomes following a third AUS implantation, a topic that has been addressed so far only in small cohorts (21). Given its comprehensive and longitudinal design, a study using the Observapur cohort has the potential to establish the first large-scale database dedicated to patients undergoing a third AUS implantation, enabling robust and meaningful analysis of this specific population.

In conclusion, this study demonstrates that a second AUS implantation can provide acceptable long-term survival. However, only a minority of patients undergoing removal of their first AUS proceed to reimplantation, and this subgroup experiences significantly lower survival outcomes compared to the better results observed after replacement procedures. Future studies should aim to elucidate the underlying reasons for these observed differences, better characterize the clinical profiles of these distinct patient populations and focus on functional outcomes that do not always correlate with device survival.

## Data Availability

Uninformed
