# Supplementary material for: Survival Without Reintervention of Second Artificial Urinary Sphincter Implants in Men: A National Healthcare Data System-Based Study in France
Source: Int Braz J Urol. 2025 Aug 30;52(1):e20250374. doi: 10.1590/S1677-5538.IBJU.2025.0374 (PMC12974964; doi:10.1590/S1677-5538.IBJU.2025.0374)

APPENDIX

Figure S1 - Description of the two distinct clinical trajectories constituting the "second AUS survival".

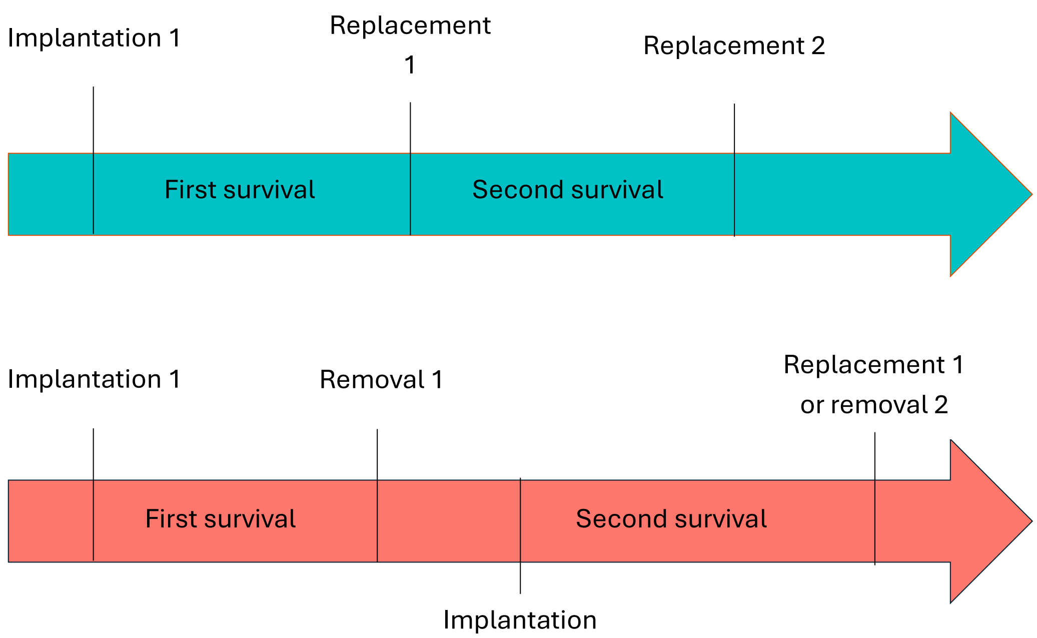

Figure S2 - Reintervention-free survival analyses stratified by center annual volume.

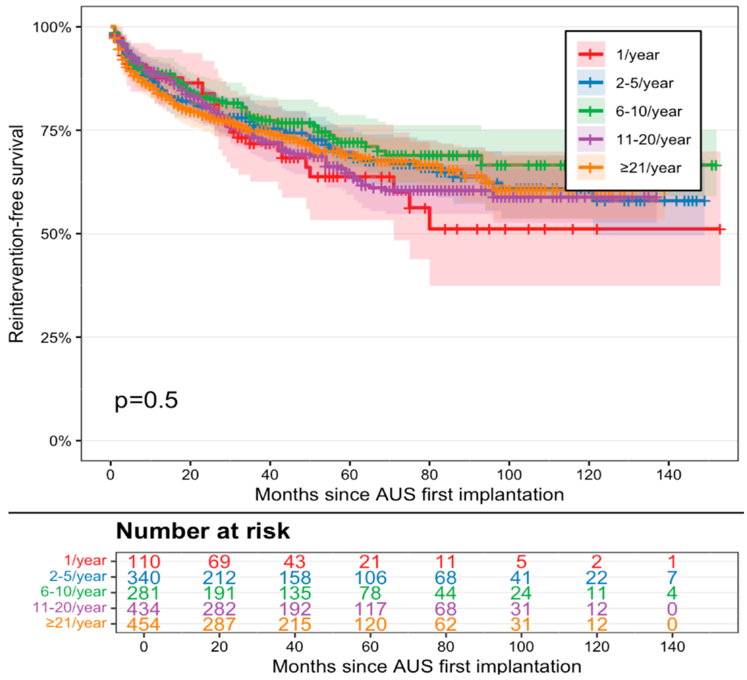

Figure S3 - Replacement-free survival analyses stratified by center annual volume.

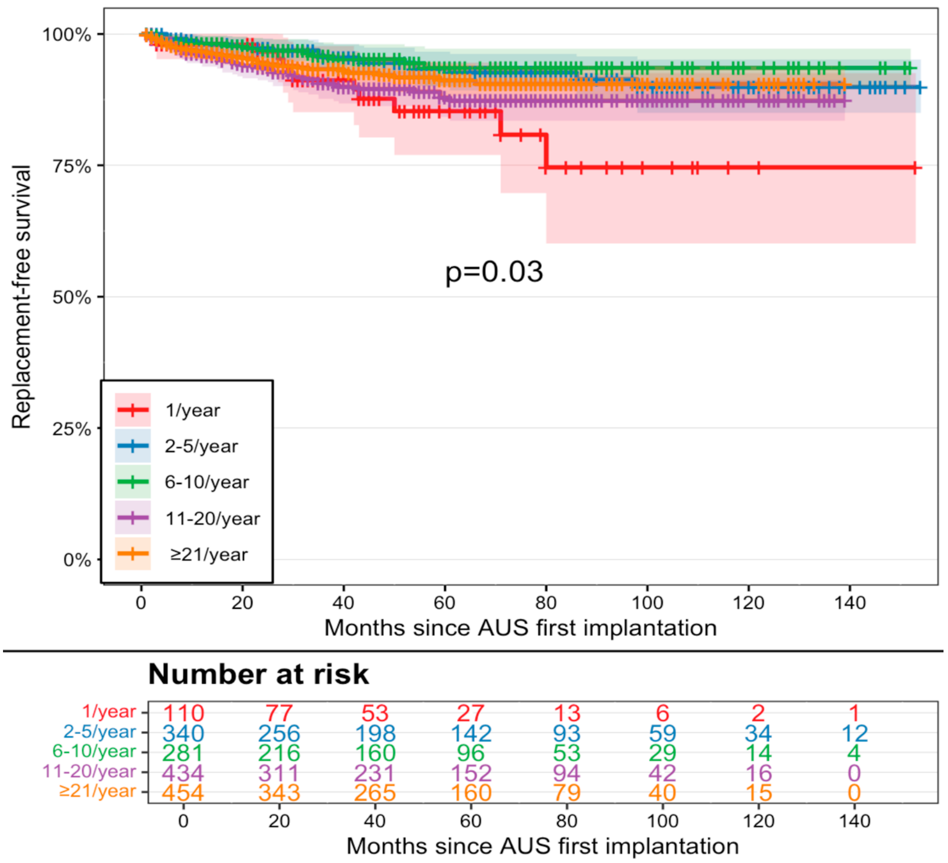

Supplement: Supplementary file 1 [file 1677-6119-ibju-52-01-e20250374-Suppl1.pdf]
